# Molecular and structural characterization of a *Bacillus cereus* strain producing an anthrax-like capsule

**DOI:** 10.1128/spectrum.00899-25

**Published:** 2025-08-13

**Authors:** Prashant Dahal, Thi Hai Au La, Md Samun Sarker, Liyanage Devthilini Fernando, Jiri Vlach, Parastoo Azadi, Michael H. Norris

**Affiliations:** 1 Pathogen Analysis and Translational Health Group, School of Life Sciences, University of Hawaiʻi at Mānoa, Honolulu, Hawaiʻi, USA; 2 Complex Carbohydrate Research Center, University of Georgia 1355, Athens, Georgia, USA; McGill University, Ste-Anne-de-Bellevue, Quebec, Canada

**Keywords:** capsule, *Bacillus cereus*, genome analysis, phylogenetic analysis

## Abstract

*Bacillus cereus* is a ubiquitous Gram-positive, spore-forming, rod-shaped saprophytic bacterium, occasionally reported to cause food-borne illnesses. However, instances of *B. cereus* strains harboring anthrax toxin and capsule genes have elevated certain strains as formidable pathogens and biothreats. This study focuses on the genomic analysis and the structural characterization of capsular material produced by the virulent *B. cereus* PATH2418 strain, isolated from the wound of a traumatic open fracture patient. The genome was sequenced using Nanopore MinION sequencing, revealing a chromosome of 5,270,283 bp and three plasmids. One plasmid, pATH1, was found to encode an operon for the biosynthesis of a bacterial capsule. This operon had sequence homology to the *Bacillus anthracis capBCADE* operon, which encodes the poly-γ-D-glutamate (PDGA) capsule. The capsule production in *B. cereus* PATH2418 was influenced by temperature and CO_2_ levels. Structural analysis of the capsular material using a combined approach of nuclear magnetic resonance (NMR) and high-performance liquid chromatography (HPLC) techniques confirmed the presence of a high-molecular-weight poly-γ-glutamate capsule, with an enantiomeric composition of approximately 67% D-glutamic acid and 33% L-glutamic acid, matching that of *B. anthracis*.

**IMPORTANCE:**

This research provides genomic characterization and phylogenetic analysis of a *Bacillus cereus* strain producing a peptide capsule. Bacteriological techniques confirmed the conditions that induce the production of this capsule. Isolation of the capsule showed that it is high-molecular-weight and proteinase-resistant. The molecular structure of the capsule was characterized using spectroscopic and enantiomeric methods. The results revealed the absence of toxin genes; however, the presence of a poly-γ-glutamic acid (PGA) capsule with a high concentration of D-glutamic acid, akin to the poly-γ-D-glutamic acid (PDGA) capsule produced by *B. anthracis*, indicates that this strain can be a surrogate for studies of the anthrax PDGA capsule. The findings also contribute to the broader knowledge of *B. cereus* as a potential biothreat, demanding the development of effective diagnostic, prevention, and treatment measures.

## INTRODUCTION

*Bacillus cereus* is a Gram-positive, endospore-forming, facultatively anaerobic, saprophyte, and opportunistic pathogen notorious for inducing food-borne illnesses (1–5). Commonly found in soil, sediments, water bodies (both fresh and marine), and even as commensals in the digestive tract of a wide range of insects, food contamination by *B. cereus* is common (2, 6). The organism can cause food spoilage as well as several forms of food-borne intoxications that are mainly self-limiting emetic or diarrheal diseases (7). *Bacillus cereus* is known to cause 1.4%–12% of total food-borne illnesses worldwide (3). It is also linked to several severe non-food-borne infections, such as bacteremia, septicemia, respiratory tract infections, neuroinvasive diseases, meningitis, and cutaneous infection among others (6, 8–10).

Taxonomically, *B. cereus* is a member of the *Bacillus cereus* group (BCG), also known as *Bacillus cereus sensu lato* (*B. cereus* s.l.), comprising at least seven other well-characterized *Bacillus* spp., including *B. anthracis* and *Bacillus thuringiensis* (11). *Bacillus cereus* and *B. anthracis*, potential human pathogens, and *B. thuringiensis,* a well-characterized entomopathogen (12), exhibit high genetic similarity and phylogenetic relatedness characterized by highly conserved genomes with similar 16S rRNA gene sequences. Traditionally, *B. cereus* and *B. anthracis* isolates were identified based on their phenotypic characteristics, including capsule production and colony morphology. Traditionally, the production of a poly-γ-glutamic acid (PGA) capsule containing mostly poly-γ-D-glutamic acid (PDGA) was used to rule out *B. cereus* from *B. anthracis* in clinical isolates (13). However, these methods have lost their importance after recent reports of *B. cereus* strains producing capsules similar to *B. anthracis* (11, 14–16).

*Bacillus anthracis* virulence is associated with its two virulence plasmids, pXO1 and pXO2. The *pagA*, *lef*, and *cya* genes on the pXO1 plasmid encode protective antigen (PA), lethal factor (LF), and edema factor (EF) which are the three components of tripartite anthrax toxin, respectively. Similarly, the *capBCADE* operon on the pXO2 plasmid encodes for biosynthetic genes of the PGA capsule, under the regulation of the *trans*-acting regulatory gene *acpA* (17). Until the early 21st century, the pXO1 and pXO2 plasmids were thought to be conserved within the *B. anthracis* lineage, until atypical *B. cereus* strains capable of producing either PGA capsule or anthrax toxin were reported. In 2004 and 2006, three *B. cereus* strains (G9241, 03BB87, and 03BB102) associated with severe pneumonia were reported to harbor a pXO1 plasmid homolog, pBCXO1, with 99.6% similarity to pXO1. *Bacillus cereus* G9241 and 03BB87 also harbor another plasmid, pBC218, that encodes for polysaccharide capsules (1, 18, 19). *Bacillus cereus* G9898 strain, containing the pBC218 plasmid, and another pneumonia-associated *B. cereus,* strain Ecl2, harboring a pXO1-like plasmid, have also been reported (1). *Bacillus cereus* strains termed *B. cereus* biovar *anthracis* (Bcbva-CA and Bcbva-CI) were isolated from primates with anthrax-like infection in Cameroon and Côte d’Ivoire, respectively, and both contain *B. anthracis* pXO1 and pXO2 plasmid homologs (20–22). PGA capsule production has been reported in *B. thuringiensis* BGSC 4AJ1, another member of the BCG (16). Several atypical *B. cereus* strains and *B. thuringiensis* strains that produce either polysaccharide or PGA capsules have been reported, underscoring the genetic promiscuity of these transferable elements among *Bacillus* spp.

A virulent *B. cereus* strain isolated from the left arm tissue of a traumatic open fracture patient was reportedly positive for PDGA capsule production, although the methodologies used and the characteristics of the capsule provided were not described (23). The bacteriological properties, genomic sequence of the strain, and the molecular structure of its capsule remained undescribed. In this work, bioinformatics of the long-read generated sequencing determined the genomic structure and gene features relevant to capsule formation. The presence of the capsule was confirmed through India ink staining of the live organisms and Alcian blue staining of the extracted capsular material. The effects of temperature and CO_2_ on capsule production were determined. Using a combined approach of nuclear magnetic resonance (NMR) spectroscopy and high-performance liquid chromatography (HPLC) techniques, the structure and enantiomeric composition of capsular material produced by this *B. cereus* strain were determined.

## RESULTS

### Capsule production in *B. cereus* PATH2418 is influenced by temperature and CO_2_

**Fig 1:**
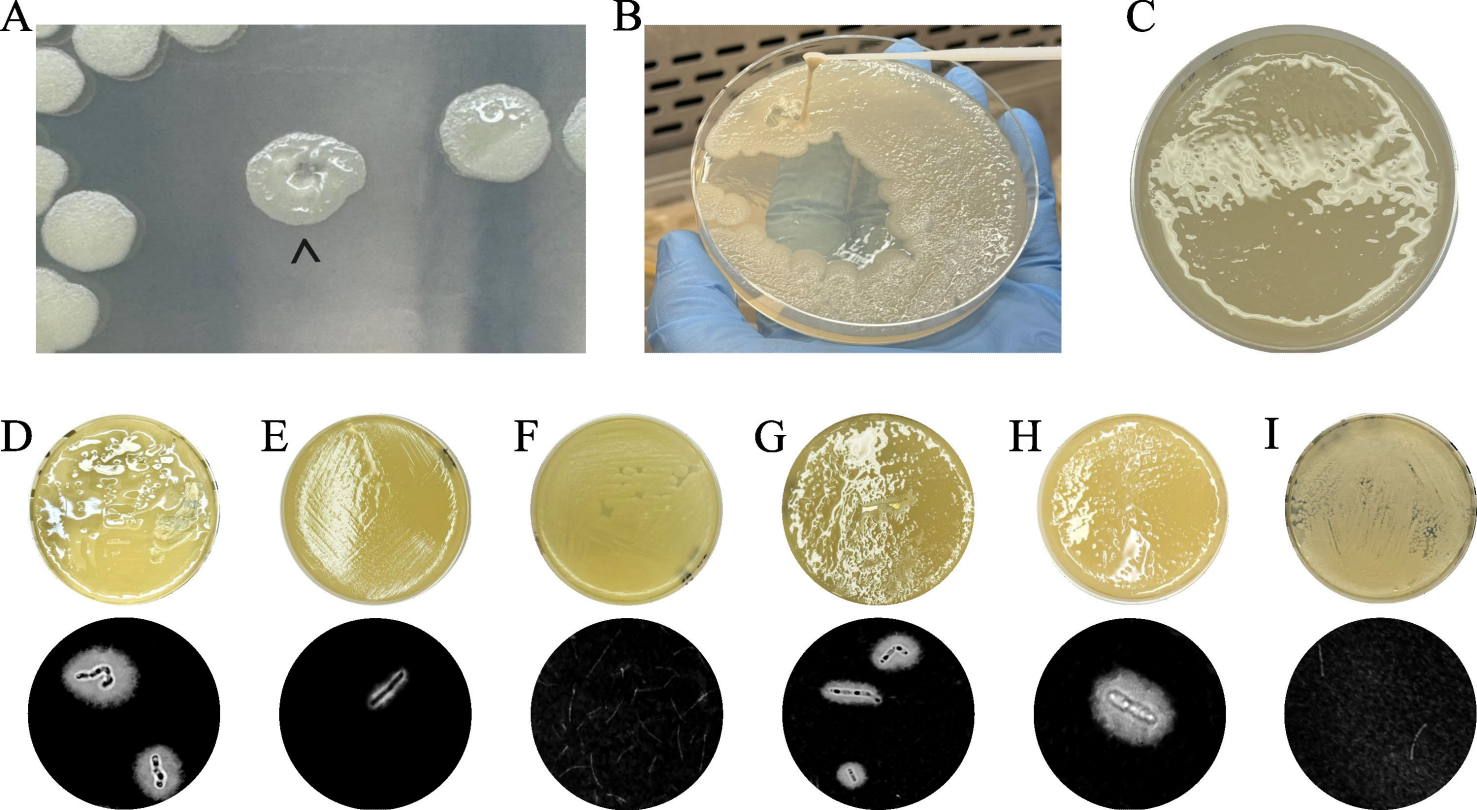
The mucoid phenotype of *Bacillus cereus* PATH2418 produced in response to CO_2_ and temperature. (**A**) Colonies were mucoid and shiny after 48 h of incubation at 30°C in air. A mucoid colony is indicated by a caret beside rough colonies and semi-rough colonies. Other colonies were non-mucoid or partially mucoid, indicating genetic instability of the phenotype. (**B**) Streak plating of the mucoid colony showed strands of shiny bacteria indicating the desired mucoid phenotype. (**C**) A glittering lawn of mucoid *B. cereus* PATH2418 on LB medium. Plates were incubated at various temperatures and atmospheric conditions: (**D**) 30°C in air, (**E**) 37°C in air, (**F**) 42°C in air, (**G**) 37°C and 5% CO_2_, (**H**) 37°C and 5% CO_2_ on NBY medium, and (**I**) acapsular *B. anthracis* Sterne at 37°C in air. Shiny plates indicate the mucoid phenotype of presumed capsule production. India ink staining (100× magnification) below each plate shows capsules as clear halos surrounding rod-shaped bacteria. *B. cereus* PATH2418 produces more capsules at lower temperatures in air, with the production decreasing as the temperature increases. In 5% CO_2_, capsule production is still evident at 37°C. (**H**) In bicarbonate medium (NYB), capsule production is more significant. (**I**) Negative control showing acapsular *B. anthracis* Sterne strain.

Plating of *B. cereus* Tor 16585 yielded a combination of dry and mucoid colonies when streaked on LB medium and incubated at 37°C, indicating a genetically unstable phenotype. From the resulting mixed colonies, a mucoid colony was isolated and saved as a pure culture, termed *B. cereus* PATH2418, for further analysis. When *B. cereus* PATH2418 was streaked and incubated at 30°C, all colonies exhibited shiny, mucoid growth, indicating the production of the capsule and stability of the phenotype in this pure culture (Fig. 1).

*B. cereus* PATH2418 was spread on LB medium and incubated at temperatures of 30°C, 37°C, and 42°C for 48 h to assess the effect of temperature on capsule production. To investigate the influence of CO_2_ and bicarbonate on capsule production, *B. cereus* PATH2418 strain was spread on LB and NBY (a capsule induction media frequently used with *B. anthracis*) medium and incubated at 37°C in a 5% CO_2_ atmosphere. After 48 h of incubation, the plated lawns were phenotypically examined for mucoid growth, and India ink staining was done to visualize the capsule surrounding the bacterial cells. The capsule production was significantly affected by both temperature and CO_2_ level (Fig. 1D through I). Among the various temperatures tested for aerobic incubation, the most pronounced capsule formation occurred at 30°C (Fig. 1D). In contrast, the capsule production was markedly diminished at 37°C (Fig. 1E), and no visible capsule production was observed at 42°C (Fig. 1F). However, in the presence of 5% CO_2_, the capsule production was prominent even at 37°C, both in LB (Fig. 1G) and NBY medium (Fig. 1H), compared to the acapsular *B. anthracis* Sterne negative control (Fig. 1I).

### Isolation and structural characterization of *B. cereus* PATH2418 capsular material

**Fig 2:**
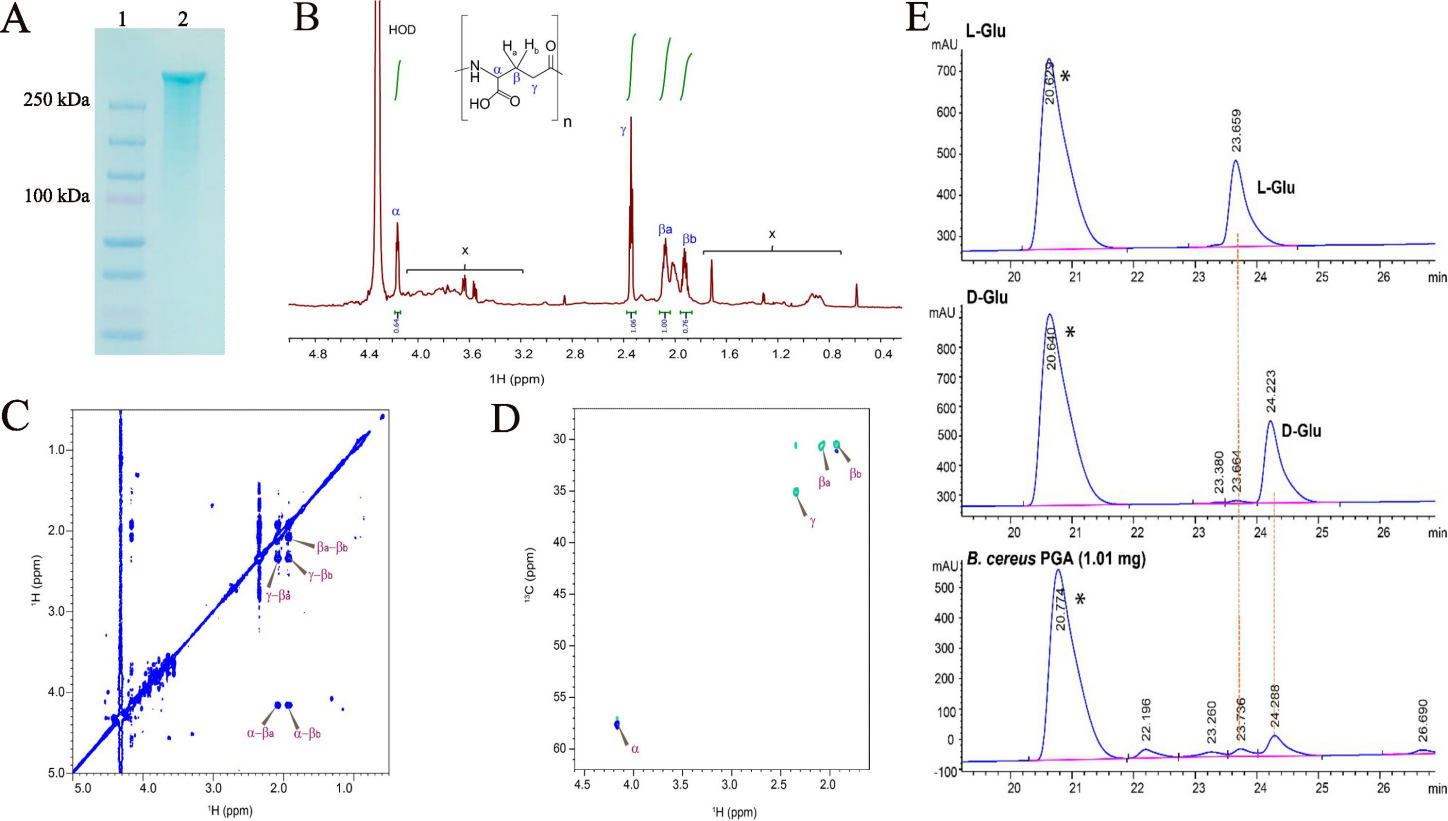
Structural and molecular analysis of capsular material from *B. cereus* PATH2418. (**A**) Capsule was extracted and purified and visualized by Alcian blue staining after polyacrylamide gel electrophoresis. Lanes: (1) Ladder showing molecular weight standard and (2) 10 ng of capsule isolated from *B. cereus* PATH2418. (**B**) The 1D ^1^H NMR result of the capsular material of *B. cereus* PATH2418 reveals the presence of poly-γ-glutamic acid. The figure represents the PGA proton signals with notable chemical shifts: α-proton at 4.16 ppm, γ-proton at 2.35 ppm, and βa and βb at 2.08 and 1.92 ppm, respectively. These distinct peaks are characteristic of γ-PGA (24). Integrals of the peaks are shown in green. The molecular structure of the γ-PGA is depicted above the spectrum. Residual water signal (HOD) is not completely shown. “x” marks the peaks of impurities, suggesting contamination with other amino acid residues from proteins or other impurities in the sample. (**C**) 2D ^1^H –^1^H correlation spectroscopy (COSY) spectrum and (D) ^13^C-^1^H heteronuclear single quantum coherence (HSQC) NMR of capsular PGA from *B. cereus* PATH2418. The signals originating from PGA are labeled in both spectra. Positive peaks (blue) in the HSQC are due to CH or CH_3_ groups, while negative peaks (cyan) are from CH_2_ groups. (**E**) HPLC chromatograms of D-glutamate, L-glutamate, and hydrolyzed *B. cereus* PATH2418 PGA capsule. About 1 mg of the sample was derivatized with S-NIFE. An asterisk indicates likely p-nitrophenol elution. The peak eluting at ~23.6 min belongs to L-glutamate, whereas the peak eluting at ~24.2 min belongs to D-glutamate. The higher peak at ~24.2 min compared to the peak at ~23.6 min indicates a higher concentration of D-glutamate than L-glutamate in the *B. cereus* PATH2418 capsular material.

The capsular material synthesized by *B. cereus* PATH2418 was extracted, purified, and fractionated using polyacrylamide gel electrophoresis. Staining the fractionated capsular material with the Alcian blue stain revealed the presence of a high-molecular-weight capsule, ranging from 50 kDa to 250 kDa (Fig. 2A).

The capsular material purified from *B. cereus* PATH2418 was analyzed using one- and two-dimensional nuclear magnetic resonance (1D and 2D ^1^H NMR). The 1D ^1^H and 2D ^1^H-^1^H COSY and ^13^C-^1^H HSQC NMR spectra confirmed the presence of poly-γ-glutamic acid as the primary component (Fig. 2B through D). The chemical shift assignments (Table S4) were consistent with the structure of γ-PGA and previously reported values (24, 25). The signal of the α-proton of the capsule appeared at 4.16 ppm, and the β-methylene protons appeared at 2.08 and 1.92 ppm, while the signals of γ-methylene protons were at 2.35 ppm. The positions of β and γ resonances were characteristic of the γ-amide linkages formed between the α-amino and γ-carboxyl groups of glutamic acid monomers, confirming the polymeric structure of γ-PGA (24, 25).

### Enantiomeric analysis of the glutamic acid present in the *B. cereus* PATH2418 capsule

To determine the absolute configuration of glutamate residues that formed the PGA capsule of *B. cereus* PATH2418, derivatization of hydrolyzed PGA with (*S*)-*N*-(4-nitrophenoxy­carbonyl) phenylalanine methoxyethyl ester (S-NIFE) was used. This formed diastereomeric urea derivatives with amino acids through nucleophilic substitution of carbamate, releasing 4-nitrophenol. The reaction products were then analyzed using HPLC, which revealed that the *B. cereus* PATH2418 capsular PGA sample contained approximately 60–75 mol% D-Glu and 40–25 mol% L-Glu, based on two independent analyses (Fig. 2E and Table 1). Standard D-Glu and L-Glu samples were prepared under identical hydrolysis conditions as the PGA sample. Two independent PGA sample analyses yielded an average molar composition of 67% (SD = 11%) D-Glu and 33% (SD = 11%) L-Glu, indicating that the γ-PGA capsule consisted mainly of D-Glu.

**TABLE 1:** Content of glutamate enantiomers in the *B. cereus* PATH2418 PGA^*a*^

Sample	D-form (mol%)	L-form (mol%)
D-Glu (standard)	96.9	3.0
L-Glu (standard)	0.0	100.0
*B. cereus* PATH2418 PGA (1.01 mg) *B. cereus* PATH2418 PGA (0.37 mg)	75.3 59.3	24.7 40.7
Mean	67% (SD = 11%)	33% (SD = 11%)

^*a*^ SD = Standard deviation.

### Bioinformatic analysis of the *B. cereus* PATH2418 genome

**Fig 3:**
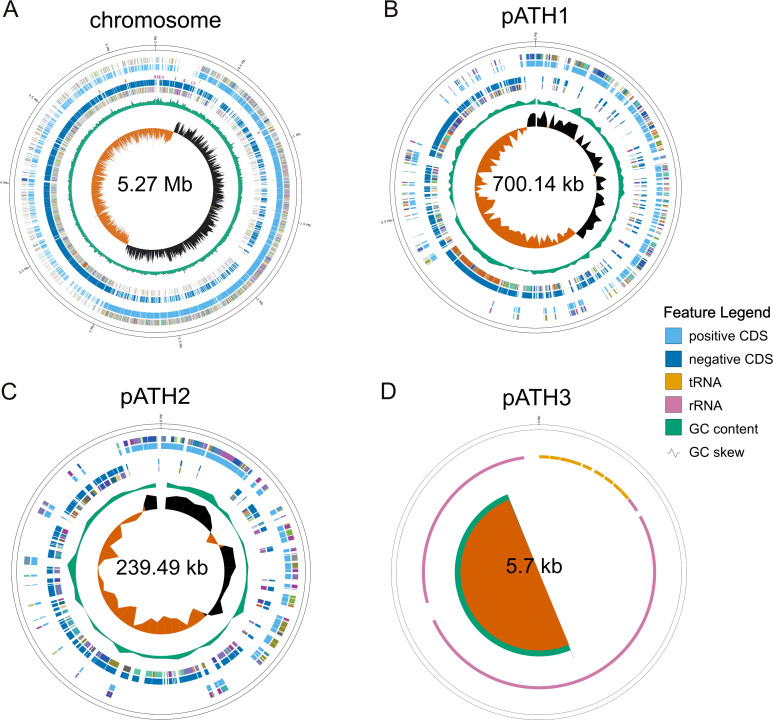
Graphical representation of the *B. cereus* PATH2418 genome. (**A**) The single circular chromosome of 5.27 Mbp present in this strain. (**B**) The largest plasmid, pATH1, of 700 Kbp. (**C**) The second largest plasmid, pATH2, of 239 Kbp. (**D**) The smallest plasmid, pATH3, at 5.7 Kbp. From outside to inside, each ring represents: Contigs; COGs on the forward strands; CDS, tRNAs, and rRNAs on the forward strands; CDS, tRNAs, and rRNAs on the reverse strands; COGs on the reverse strands; GC content; GC skew.

Genome characteristics of *B. cereus* PATH2418 are summarized in Table 2. The assembly yielded four contigs with an N_50_ of 5,270,283 bp, the largest of which was a complete, closed chromosome measuring 5,270,283 bp. The overall GC content of the assembly was 34.9%. Analysis of the sequencing data indicated that the *B. cereus* PATH2418 strain comprises one large chromosome of 5,270,283 bp (Fig. 3A) and three plasmids, dubbed pATH1, pATH2, and pATH3, of varying sizes (Fig. 3B through D). Plasmid pATH1 was the largest plasmid, measuring 700,138 bp, while pATH2 was 239,493 bp. The pATH3 was the smallest, with a length of 5,700 bp. The chromosome and two plasmids, pATH1 and pATH2, had lower GC content of 32–35%. In contrast, the pATH3 had a higher GC content of 51%. The genome was predicted to contain 6,339 CDS, 45 rRNA genes, and 115 tRNA genes. While the elevated GC content of pATH3 initially raised the possibility of horizontal gene transfer from a distantly related organism, the BLASTN analysis revealed that sequences within pATH3 share high homology with chromosomal regions of *Bacillus *spp. of the *Bacillus cereus* group and encodes only rRNA and tRNA. This suggests that pATH3 may have originated through the mobilization or rearrangement of native chromosomal elements, such as insertion sequences or possibly phage.

**TABLE 2:** Genomic characteristics of *B. cereus* PATH2418

Features	*B. cereus* PATH2418			
	Chromosome	pATH1	pATH2	pATH3
Size (bp)	5,270,283	700,138	239,493	5,700
No. of CDS	5,394	702	243	0
% G + C content	35.30%	32.26%	33.26%	51.42%
rRNA	42	0	0	3
tRNAs	106	2	0	9
sRNAs	0	0	0	0
No. of repeat regions	49	18	9	0
Protein-encoding genes with functional assignment	3,386	283	90	0
Protein-encoding genes without functional assignment	2,008	419	153	0
No. of proteins with functional assignment	4,205	337	121	0
Hypothetical proteins	1,189	365	122	0
No. of antibiotic resistance genes (PATRIC source)	48	3	1	0
No. of virulence factor-encoding genes	11	1	0	0

**Fig 4:**
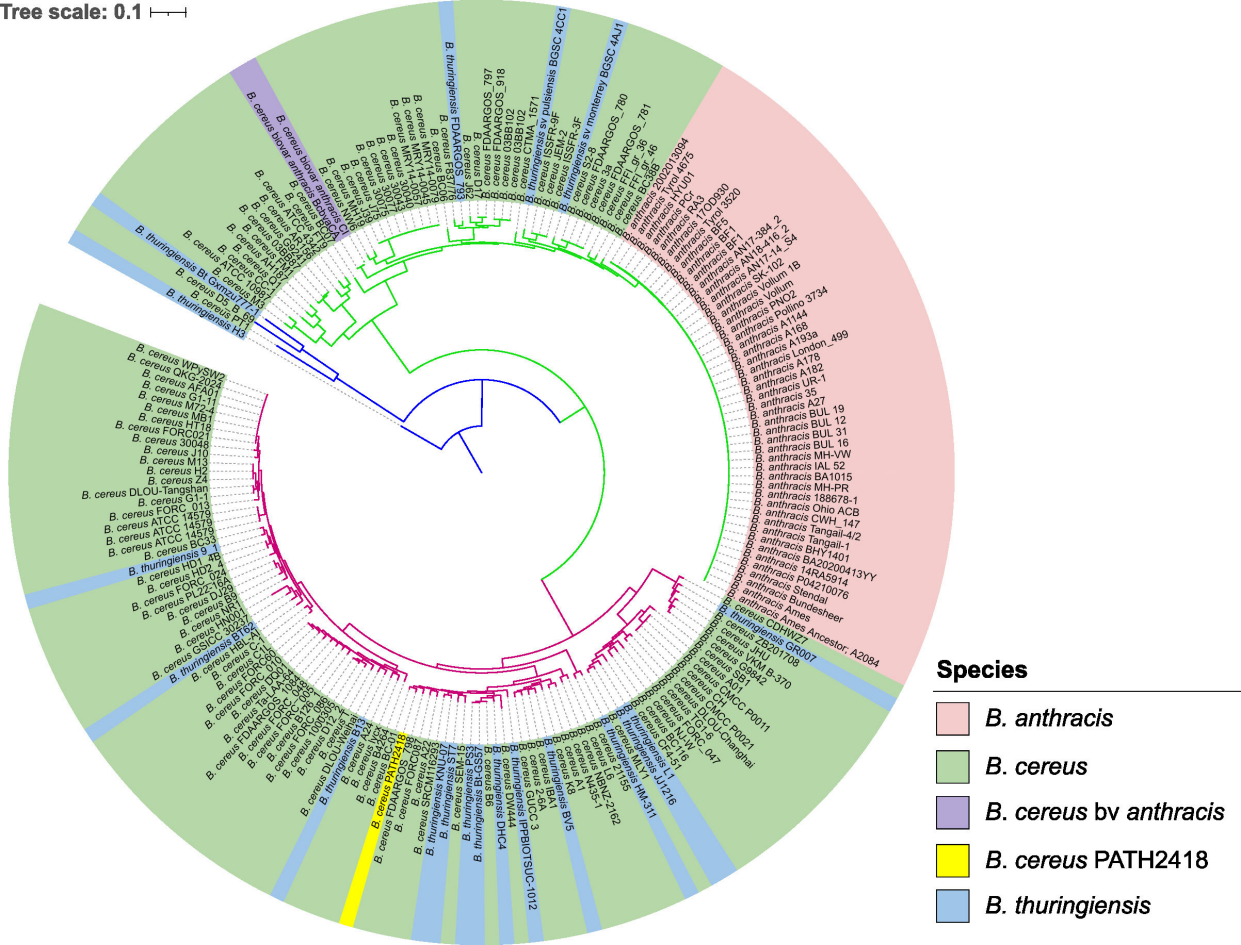
Maximum likelihood tree of chromosomal SNP alignments. *Bacillus cereus* PATH2418 strain from this study (yellow background) was compared to other *B. cereus* group species: *B. cereus* (green background), Bcbva (purple background), *B. anthracis* (red background), and *B. thuringiensis* (blue background). The different colored branches indicate the main clades containing pathogenic *Bacillus*.

The phylogenetic relationships of the whole-genome of *B. cereus* PATH2418 with other *Bacillus* species were examined based on single-nucleotide polymorphism (SNP). Complete genomes of 126 *B. cereus*, 19 *B. thuringiensis*, 2 *B. cereus* bv *anthracis*, and 48 *B. anthracis* strains whose metadata (isolation source and location) was available were retrieved from NCBI (the accession number and strain names are compiled in Table S1). The analysis revealed that the *B. cereus* PATH2418 is closely related to the *B. cereus* BC-01 (Fig. 4) and not to other *B. cereus* that are closely related to *B. anthracis*, such as Bcbva strains.

**Fig 5:**
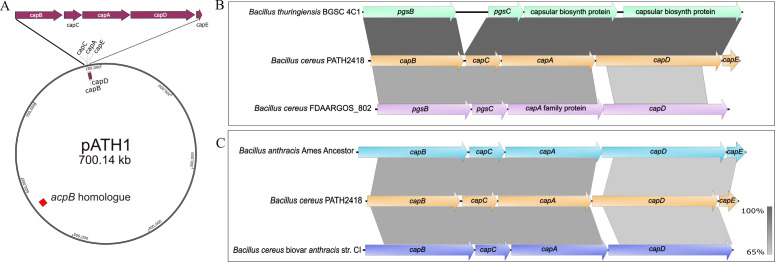
Location of the *cap* operon on pATH1 and its nucleotide identity with other *Bacillus* capsule synthesis operons. Plasmid pATH1 contains PGA capsule operon which aligns closely with the PGA capsule operon of *Bacillus thuringiensis* BGSC 4C1. (**A**) Graphical representation of the pATH1 with PGA capsule biosynthetic operon. The five genes involved in capsule production are *capA*, *capB*, *capC*, *capD*, and *capE*. An *acpB* homolog, which is a capsule operon transcriptional regulator found in *B. anthracis* Ames, is highlighted in red. (**B**) The alignment of the PGA capsule operon of *B. cereus* PATH2418 (orange) with the capsule operon of *B. thuringiensis* BGSC 4C1 (mint green) and *B. cereus* FDAARGOS_802 (lavender). In the upper section, all four genes in the *cap* operon of *B. thuringiensis* BGSC 4C1 aligned with the predicted operon of *B. cereus* PATH2418. In the lower section, the *pgsB*, *pgsC*, *capA* family protein-encoding gene, and *capD* of *B. cereus* FDAARGOS_802 aligned with the *cap* operon in *B. cereus* PATH2418. (**C**) The alignment of the predicted capsule operon of *B. cereus* PATH2418 (orange) with the *cap* operon of *B. anthracis* Ames Ancestor (cyan) and Bcbva CI (blue). In the upper section, all five genes in the *cap* operon of *B. anthracis* Ames Acestor aligned with the predicted operon of *B. cereus* PATH2418. In the lower section, all four genes in the *cap* operon of Bcbva CI (Bcbva CI lacks a *capE* homologue) aligned with the *cap* operon in *B. cereus* PATH2418. The gradient scale bar corresponds to percent identity at the nucleotide level, based on BLAST results used in EasyFig. Darker shading indicates higher nucleotide identity between corresponding genes, whereas lighter shading indicates lower identity.

The genomic analysis demonstrated that the largest plasmid, designated as pATH1, of the *B. cereus* PATH2418 encoded a region similar to the poly-γ-glutamic acid capsule-encoding operon, the *capBCADE* operon of *B. anthracis*, along with a fragment of the capsule regulator, *acpB* regulator, of *B. anthracis* pXO2 plasmid (Fig. 5A). The PGA capsule operon of *B. cereus* PATH2418 also harbors five genes homologous to the *capBCADE* operon of *B. anthracis*. NMR spectroscopy and HPLC analysis corroborated that the capsular material in *B. cereus* PATH2418 is poly-γ-glutamic acid, predominantly composed of poly-γ-D-glutamic acid. The synthesized PGA in *B. cereus* PATH2418 is anchored to the bacterial surface, forming a visible capsule, as evident in India ink preparations (Fig. 1), like in *B. anthracis*. Accordingly, the genes within the *B. cereus* PATH2418 PGA operon are designated as *capB*, *capC*, *capA*, *capD*, and *capE*, consistent with the nomenclature used in the *B. anthracis* for its PGA capsule operon (26).

The PGA capsule operon of *B. cereus* PATH2418 spans 4,640 bp (nucleotide positions: 81,647–86,286) in pATH1 with a GC content of 33% and comprises five genes: *capB*, *capC*, *capD*, *capA, *and *capE*, which are arranged sequentially. The genomic features and predicted function of each gene in the *cap* operon are summarized in Table 2.

The poly-γ-glutamate capsule operon serves as a major virulence factor of *B. anthracis*, yet it is also reported in some strains of other species within the *Bacillus cereus* group. However, the number of genes and the degree of genetic similarity within the capsule operon exhibit varies among the members of this group. To understand the similarities, at the nucleotide level, of the PGA capsule operon of *B. cereus* PATH2418, a BLAST analysis was performed using the NCBI BLASTN tool. The capsule operon of *B. cereus* PATH2418 was compared to the capsule operons of *B. thuringiensis* BGSC4C1 (Accession no. CP015177.1), *B. cereus* FDAAGROS_802 (Accession no. CP053962.1), Bcbva str. CI (Accession no. CP001748.1), and *B. anthracis* Ames Ancestor (Accession no. AE017335.3) (Fig. 5B and C). The BLASTN results were visualized using Easyfig 2.2.5 (27). The analysis indicated that the capsule operon of *B. cereus* PATH2418 is more closely related to that of *B. thuringiensis* BGSC 4C1 than to other *Bacillus* spp. used in the analysis (Fig. 5B and C).

In addition to BLASTN, a BLAST analysis of the amino acid sequences was performed for each gene in the operon against the non-reductant GenBank CDS translation, PDB, SwissProt, PIR, and PRF databases, excluding environmental samples from WGS projects, using the NCBI BLASTP program (28, 29). Comparative analyses of nucleotide and amino acid sequences of the *cap* genes and corresponding Cap proteins from *B. cereus* PATH2418 and PGA encoding other *Bacillus* spp. are delineated in Table 3 and Table 4, respectively.

**TABLE 3:** Genomic features and predicted functions of capsule-associated genes in the *cap* operon of *B. cereus* PATH2418 (pATH1)

Gene	Length (in bp)	Nucleotide position	Protein encoded	Protein size (kDa)	Number of amino acids	Predicted function of proteins
*capB*	1,185	81,647–82,831	CapB	44.2	394	(Polyglutamate polymerase) peptide synthase activity for γ-PGA synthesis (30, 31)
*capC*	450	82,860–83,309	CapC	16.5	149	(Polyglutamate polymerase) peptide synthase activity for PGA synthesis (30) Supplying γ-glutamyl units to *capB*/*pgsB* (31)
*capA*	1,191	83,320–84,510	CapA	45.2	396	Transport of PGA through the membrane (30)
*capD*	1,602	84,523–86,124	CapD	59.6	533	Depolymerize larger capsule polymers into smaller glutamic acid peptide fragments (30, 32, 33) Covalent anchoring of polyglutamate to peptidoglycan (30, 32)
*capE*	141	86,146–86,286	CapE	5.3	46	Transport of PGA through the membrane (34)

**TABLE 4:** Nucleotide and amino acid identity of *cap* genes of *B. cereus* PATH2418 with *cap* homologs in related *Bacillus* spp^*a*^

Gene	Nucleotide identity (%)				Amino acid identity (%)			
	*Ba*	Bcbva	*Bc*	*Bt*	*Ba*	Bcbva	*Bc*	*Bt*
*capB*	76.04	76.04	76.07	98.31	78.41	78.41	78.66	98.73
*capC*	81.11	81.33	81.78	99.56	83.22	83.89	83.89	99.33
*capA*	72.43	72.53	72.31	97.98	61.11	61.11	61.27	90.91
*capD*	64.80	64.92	65.18	96.49	57.91	57.91	56.68	96.48
*capE*	70.09	NA	NA	NA	54.76	NA	NA	NA

^*a*^ *Ba*, *Bacillus anthracis* Ames Ancestor. Bcbva, *Bacillus cereus* biovar *anthracis* strain CI. *Bc*, *Bacillus cereus* FDAARGOS_802. *Bt*, *Bacillus thuringiensis* BGSC 4C1. NA, not applicable, homologous genes missing in these strains.

Similarity between the capsule operon on pATH1 of *B. cereus* PATH2418 and other capsule synthesis encoding plasmids of other *Bacillus *spp. were aligned with pXO2 and pXO2-like plasmids from *B. anthracis* Ames Ancestor, Bcbva CI, *B. cereus* FDAARGOS_802, and *B. cereus* 03BB108 using the Mauve alignment tool. The alignment revealed that the capsule regulator on pATH1 (homologous to *B. anthracis acpB*) is located much farther from the capsule operon compared to the tested strains (Fig. S1). The *acpB* homolog is approximately 219 kb upstream of the *capB* gene in this plasmid, whereas it is only 6.6 kb downstream of the *capB* gene in the pXO2 plasmid of *B. anthracis* Ames Ancestor.

To ascertain the genetic relatedness of pATH1 from *B. cereus* PATH2418, a SNP-based phylogenetic analysis was visualized using the PhaME, comparing the pATH1 with PGA-encoding plasmids from *B. cereus* FDAARGOS_802 (Accession no. CP053962.1), *B. cereus* 03BB108 (Accession no. CP009636.1), *B. thuringiensis* (Accession no. CP015177.1), Bcbva CI (Accession no. CP001748.1), and pXO2 plasmids of 26 additional *B. anthracis* strains (accession number and strain name in Table S2). The analysis revealed that pATH1 of *B. cereus* PATH2418 exhibits a close genetic relation with *a B. thuringiensis* BGSC 4C1 plasmid, and had some degree of relatedness to the plasmids of *B. cereus* FDAARGOS_802 and *B. cereus* 03BB108. In contrast, it was found to be distantly related to pXO2 and pCIXO2 plasmids (Fig. S2).

## DISCUSSION

Poly-γ-glutamic acid (PGA) is produced by certain Gram-positive bacteria, particularly the members of the Bacilli class within the Bacillales order (26) in the form of a capsule or slime layer. In many *Bacillus *spp., including *B. subtilis*, *B. licheniformis*, and *B. megaterium*, the *capD* gene is absent. Hence, they are unable to form distinct, anchored capsules. Rather, they produce a PGA slime layer surrounding their cells. *B. anthracis*, however, contains a functional *capD* gene in the *capBCADE* gene operon in the pXO2 plasmid, which anchors the PGA to the meso-diaminopimelic acid (DAP) of the peptidoglycan of the bacterial cell wall and forms a stable PGA capsule (32, 35). The presence of the PGA capsule was previously thought to be unique to *B. anthracis*; however, other closely related species with PGA capsule have been identified. Detection of pXO2 plasmid homologs in *B. cereus* and *B. thuringiensis* using PCR-based approaches shows that pXO2-like plasmids are distributed in these species (36, 37).

The *B. cereus* PATH2418 strain studied in this project contains the PGA capsule-encoding operon. Although the plasmid containing the capsule operon exhibited limited similarity to the pXO2 plasmids of *B. anthracis* strains, it shows a high degree of similarity to the PGA capsule-encoding plasmids of *B. thuringiensis* BGSC 4C1 and *B. cereus* FDAARGOS_802 and 03BB108 strains. All four capsule-encoding genes of *B. thuringiensis* BGSC 4C1 had high nucleotide similarity, approximately 98%, with the corresponding genes in the *cap* operon of pATH1, with the exception of the *capE* gene. All five *cap* genes from the *capBCADE* operon of pATH1 aligned with the five *cap* genes in the *capBCADE* operon of the pXO2 plasmid in *B. anthracis* Ames Ancestor, exhibiting a nucleotide similarity of about 74%—lower than the *B. thuringiensis*. A similar result, about 74% nucleotide similarity, was found between the *capBCAD* genes of Bcbva str. CI and the *cap* operon of pATH1. Regarding the PGA capsule operon of *B. cereus* FDAARGOS_802, only the four genes* capB*, *capC*, *capA*, and *capD* aligned with approximately 75% nucleotide similarity. Though this plasmid contains all 5 genes of the *cap* operon, the capsule regulator of pATH1 (the *acpB* gene) was located much farther from the capsule operon compared to that of *B. anthracis* and Bcbva. Another notable feature about *B. cereus* PATH2418 pATH1 is that at ~700 kbp it is much larger than other known PGA capsule-encoding plasmids of *B. anthracis* (~96 Kb), *B. cereus* FDAARGOS_802 (~282 Kb), Bcbva CI (~94.5 Kb), and *B. thuringiensis* BGSC 4C1 (~267.6 Kb).

The information about the molecular structure of the PGA capsule encoded by *B. thuringiensis* BGSC 4C1 and *B. cereus* FDAARGOS_802 is not available, so our ability to directly compare the structure of the PGA capsule in *B. cereus* PATH2418 with that of other *Bacillus* is limited. However, it is important to note that the structure of the PGA capsule in *B. cereus* PATH2418 is very similar to that of *B. anthracis*. A key distinction is that the PGA capsule of *B. cereus* PATH2418 consists of a mixture of D- and L-isomers of glutamic acid, ~67% and ~33%, respectively, whereas the PGA capsule of *B. anthracis* is composed solely of D-isomers (38, 39). The NMR analysis also indicated protein impurities, which may have contributed additional L-Glu and led to underestimation of the true D-Glu content in *B. cereus* PATH241 capsular material. Racemization during the hydrolysis step was minimal, given the low content of L-Glu in the D-Glu standard (Table 1), and likely did not significantly affect the D/L content determination.

*B. cereus* PATH2418 is not the first *B. cereus* strain reported to produce PGA capsule. It has also been identified in Bcbva strains, as well as in closely related strains JF3964 and BC-AK (6). *B. cereus* strains, such as 03BB102 and 03BB108, are also known to have *cap* genes but are unable to produce a PGA capsule (19). Seven atypical pathogenic *B. cereus* strains—namely G9241, 03BB87, 03BB102, Elc2, FL2013, LA2007, and G9898—carry plasmids encoding the biosynthesis of a functional hyaluronic acid capsule. Of these, G9241, 03BB87, LA2007, and G9898 also produce a polysaccharide capsule (6). BLAST analysis revealed no *B. cereus* PATH2418 homologues of the hyaluronic acid capsule operon (*hasACB*) or the exopolysaccharide capsule operon (*bpsX-H*) found in *B. cereus* G9241 (40), suggesting that it predominantly produces a PGA capsule. These atypical strains of *B. cereus* produce capsules, either independently or alongside anthrax-like toxins, and have been implicated in severe pneumonia and anthrax-like infection in humans and other mammals (1, 6). Capsule is regarded as a critical virulence determinant in encapsulated pathogenic bacteria, primarily due to its role in immune evasion. The PGA capsule of *B. anthracis* is well documented for its role in protecting vegetative cells from phagocyte killing, whether mediated by complement-dependent or complement-independent mechanisms (41). Similarly, the polysaccharide capsule of *B. cereus* G9241, in conjunction with anthrax toxin component PA, has been implicated in anthrax-like infection (42). In the case of *B. cereus* PATH2418, the presence of the PGA capsule may likewise contribute to resistance against phagocytosis and complement binding, thereby enhancing its pathogenic potential, together with other unknown virulence factors, despite an absence of anthrax toxin genes and their homologues.

We saw that capsule production is affected by growth temperature and atmosphere. Capsule production was enhanced at 30°C in ambient atmosphere but was reduced at 37°C and was imperceptible at 42°C by India ink staining. Growth in 5% CO_2_ with or without bicarbonate, at 37°C, increased capsule production to the level exhibited at 30°C in the ambient atmosphere (Fig. 1). The observed increase in capsule production under 5% CO_2_ aligns with findings in *B. anthracis*, where elevated CO_2_ and bicarbonate levels at 37°C induce expression of the virulence regulator *atxA*, thereby upregulating both toxin and capsule genes (21, 43). Although a homolog of *atxA* was not identified in the *B. cereus* PATH2418 genome, the enhanced production of the PGA capsule under elevated CO_2_ may reflect a similar adaptive response to host-like conditions. Given that mammalian blood contains a significantly higher partial pressure of CO_2_ compared to the ambient atmosphere (44), it is plausible that CO_2_ acts as a physiological signal promoting capsule biosynthesis. This may facilitate immune evasion in *B. cereus* PATHJ2418 via a regulatory mechanism that remains to be elucidated.

Recent advancements in whole-genome sequencing and the escalating use of this technology have elucidated the presence of anthrax toxin-encoding and capsule-encoding genes in many strains of *B. cereus* and *B. anthracis*. The discovery of pathogenic *Bacillus *spp., such as *B. cereus* PATH2418, possessing a functional PGA capsule operon underscores the ongoing process of gene transfer and seemingly limitless potential for horizontal gene transfer, recombination, and transformation in the environment. This is another example of potentially pathogenic strain emergence and biothreat agent evolution from less pathogenic bacterial species.

The identification of encapsulated *B. cereus* strains blurs the classical taxonomic boundaries within the *B. cereus* group, cautioning against sole reliance on phenotypic traits, such as capsule presence, for species-level identification in clinical or diagnostic contexts. The presence of the PGA capsule in multiple *B. cereus* group members undermines its specificity as a diagnostic marker for *B. anthracis*.

*B. cereus* PATH2418 harbors a *capBCADE* operon that is both syntenic with and homologous to that of *B. anthracis*, and its capsule, enriched in the D-form of glutamic acid, exhibits a high level of structural similarity to the *B. anthracis* capsule. However, due to the absence of a pXO1 homologue and anthrax toxin genes, this strain can be safely handled under BSL-2 conditions and may serve as a biosafe model for PGA capsule isolation and downstream applications.

## MATERIALS AND METHODS

### Reagents

All chemicals were purchased from Sigma-Aldrich, VWR International, or Fisher Scientific unless otherwise stated. Unless otherwise noted, solutions were prepared in ultra-pure Milli-Q water. LB medium (RPI Corp., Mount Prospect, IL, USA) was used as a culture medium during the experiment. Nutrient broth–yeast (NBY) extract medium supplemented with 0.7% NaHCO_3_ was prepared as described by Green et al. (13). Briefly, 4 g of nutrient broth (RPI Corp., Mount Prospect, IL, USA), 1.5 g of yeast extract (RPI Corp., Mount Prospect, IL, USA), and 7.5 g of agar (RPI Corp., Mount Prospect, IL, USA) were dissolved in 450 mL of Milli-Q water. pH was adjusted to 6.8 using NaOH (aqueous 5M NaOH solution), and the medium was sterilized by autoclaving. Filter-sterilized 7% NaHCO_3_ solution (50 mL) was added to the medium following autoclaving.

### Bacterial strains and growth conditions

*B. cereus* Tor 16585 strain, which was originally isolated from the left arm tissue of a human patient, was obtained from BEI resources (BEI Resources, NIAID, NIH: *Bacillus cereus*, Strain Tor 16585, NR-12151) (45). We selected the one colony with prominent capsule production, designated it as *B. cereus* PATH2418, and used it in this study. *B. cereus* PATH2418 was inoculated in LB medium and NBY bicarbonate medium and incubated at different temperatures (30°C, 37°C, and 42°C) with or without 5% CO_2_ to study capsule production patterns in different culture conditions.

### Capsule detection using India ink staining and microscopy

After 48 h of incubation under various conditions, India ink staining was used to visualize the bacterial capsule as described previously (46). Briefly, a drop of India ink (Becton, Dickson and Company, Sparks, MD, USA) was placed on a clean glass slide, and a small amount of bacterial culture was mixed into the India ink using a sterile inoculating loop. A cover slip was gently placed over the suspension, and the edges were sealed using a clear nail polish to prevent drying. The stained preparations were observed under a light microscope using a 100× oil immersion objective.

### Capsule extraction and purification

*B. cereus* PATH2418 was grown overnight (14 to 16 h) in LB broth, shaking continuously at 30°C. Following the overnight incubation, 100 µL of the stock culture was spread on each of 30 LB agar plates and incubated at 30°C for 48 h. Bacteria were harvested by scraping the culture in the presence of 2 mL of pure water using a cell scraper. The harvested cells were transferred to a 50 mL Falcon tube (15 mL in each tube), and 5 mL of pure water was added and vortexed at maximum speed for 10 min to separate the capsule from the bacterial surface and dissolve it in water. The vortexed mixture was centrifuged at 20,000 × *g* for 15 min, and the clear, viscous supernatant was transferred to another clean 50 mL Falcon tube. The supernatant was filtered through a 0.22 µm vacuum filter, and 100 µL of the filtrate was spread on an LB agar plate and incubated for 48 h to ensure sterility.

Once the filtrate was verified to be sterile, protein and nucleic acids were degraded as previously described (47, 48). Briefly, MgCl_2_ was added to a final concentration of 5 mM. DNase-I and RNase-A were added to a final concentration of 20 µg/mL each and incubated at 37°C for 2 h. Proteinase K was added to a final concentration of 30 µg/mL and incubated at 58°C overnight (~16 h). The treated solution was dialyzed against pure water in ice using a 7 kDa SnakeSkin Dialysis Tubing (Thermo Scientific, Rockford, IL, USA) for three days, changing water three times a day. The dialyzed material was lyophilized and resuspended in pure water at a final concentration of 10 mg/mL and stored at −80°C.

### Capsule visualization

Ten nanograms of the lyophilized capsule (1 µL) was mixed with 5 µL of SDS gel loading dye (BIO-RAD 4× Laemmli Sample Buffer), and 14 µL of pure water was added to it. The solution was heated at 90°C for 10 min in a heat block, and the solution was loaded into SDS gel (MP Biomedicals Precast Gel Plus, Tris-Gly, 4 to 20%) and run for 90 min at 120 V.

Alcian blue staining was performed to visualize the capsule. Simply, after electrophoresis, the gel was washed with ultra-pure water for 2 h, with the water changed every 30 min in an orbital shaker at 40 RPM. 0.1% Alcian blue stain in 3% acetic acid solution was added to the washed gel and allowed to stain for 1 h with continuous shaking. Destaining was done for 2 h using a 3% (vol/vol) acetic acid solution and Kimwipes, with the solution changed every 30 min. The gel was left in ultra-pure water overnight and visualized the next day under transillumination.

### NMR spectroscopy of the capsule

About 0.5 mg of the capsule sample was dissolved in 200 µL D_2_O (99.96% D) and lyophilized. Then, the sample was dissolved in 50 µL D_2_O (99.96% D) with 0.5 µL 50 mM DSS-d_6_ (hexadeuterio-sodium trimethylsilylpropanesulfonate) and transferred to a 1.7 mm NMR tube for NMR analysis. NMR data were acquired at 343 K on a Bruker NEO spectrometer (^1^H, 800 MHz) equipped with a TCI cryoprobe using standard pulse sequences. The acquisition parameters are summarized in Table 5. Chemical shifts were referenced to DSS (δ_H_ = 0 ppm, δ_C_ = 0 ppm). The spectra were processed and analyzed with *MestReNova* v14.2.1-27684.

**TABLE 5:** Acquisition parameters

	Acquisition parameters										
Experiment	Temp (K)	ω_0, 1H_ (MHz)	d1(s)	NS	td2	td1	aq2 (s)	aq1(s)	sw2(ppm)	sw1 (ppm)	Exp. Time
1D ^1^H	343	800	1.5	8	50,000	–^*a*^	2.6	–	16	–	52 s
2D COSY			1.5	8	2,048	256	0.16	0.05	7	6.5	58 min
2D HSQC			1.5	16	2,048	256	0.1	0.005	12	120	1.50 h

^*a*^ – indicates not applicable.

### Hydrolysis, S-NIFE derivatization, and enantiomeric analysis of the capsule

To distinguish between the D- and L-enantiomers of glutamate residues that form the PGA, S-NIFE derivatization of hydrolyzed PGA was used as described previously (49). Approximately 1 mg of D-glutamic acid (Sigma-Aldrich), L-glutamic acid (Sigma-Aldrich), and the PGA sample were each dissolved in 1 mL of 6 M HCl in a 14 mL glass screw-cap tube. The tubes were sealed with Teflon-lined caps and incubated in a heating block set at 170°C for 30 min to minimize racemization (50). Cooled samples were evaporated to dryness with a stream of nitrogen, put in a vacuum for 3 h, and dissolved in deionized water to a concentration of 1 mg/mL.

To 25 µL of hydrolyzed sample, 0.5 µL triethylamine and 25 µL (*S*)-*N*-(4-nitrophenoxy carbonyl) phenylalanine methoxyethyl ester (S-NIFE, 5 mg/mL in acetonitrile) were added, and the mixture was incubated for 25 min at room temperature. The reaction was quenched by adding 200 µL of 0.1% trifluoroacetic acid (TFA) in water.

The reaction products (25 µL load) were separated on an Agilent C18 column (25 cm × 4.6 mm, 5 µm) attached to a high-performance liquid chromatography system (Agilent 1260 Infinity II), using a linear elution gradient from 5% to 90% (vol/vol) acetonitrile in water containing 0.1% TFA over 90 min at 1 mL/min flow rate. Eluting species were detected by recording the absorbance at 205 nm.

### Nucleic acid extraction and whole-genome sequencing

Nucleic acids were extracted using QIAamp BiOstic Bacteremia DNA Kit according to the manufacturer’s instructions. Briefly, *B. cereus* PATH2418 was grown overnight in 3 mL of LB broth at 37^°^C. Five hundred microliters of the overnight culture was centrifuged at 13,000 × *g* for 2 min to pellet the bacteria, and the manufacturer’s protocol was followed to isolate the nucleic acids.

The library was prepared following the protocol provided by Oxford Nanopore Technologies (ONT) using the Native Barcoding Kit 24 V14 (SQK-NBD114.24) from the ONT. All procedures were performed on ice, except for the incubation steps, which needed different temperatures as mentioned in the protocol. Briefly, ~400 ng of the genomic DNA was repaired and end-prepped in the DNA repair and end-prep step, following the ONT protocol before ligating the native barcode in the native barcode ligation step. The adaptor was ligated to the barcoded gDNA library in the adaptor ligation and clean-up step as instructed. The purified library was eluted in a 15 µL elution buffer. Twelve microliters of the gDNA library was mixed with sequencing buffer (37.5 µL) and library beads (25.5 µL) to bring the sample to a total volume of 75 µL. This complete library was loaded in the MinION flow cell (R10.4.1), and the sequencing was done in the MinION Mk 1B sequencing device using the MinKNOW software (v24.06.5) in the high accuracy basecall configuration. The fastq file was submitted to the NIH Sequence Read Archive under BioProject Accession PRJNA1241850 and sample Accession SAMN47572344, and the assembled genome is available in GenBank at accession numbers CP196405–CP196408.

### Bioinformatics and genome analysis

Raw data were processed on the University of Hawaiʻi KOA High Performance Computing Cluster. The Nanopore raw reads were *de novo* assembled using Flye (v2.9.5) (51). The assembly was assessed by the Quality Assessment Tool for Genome Assemblies (QUAST) (usegalaxy.org/), and the assembled genome was annotated using Prokka (v1.14.6) (52) and BLASTed against the *Bacillus cereus* group. The assembled contigs were visualized using GenoVi, an automated circular genome visualizer tool (53). The contigs were analyzed using the BV-BRC comprehensive genome analysis (54) web resource to gather information about the different kinds of genes encoded in the chromosomes and plasmids.

Whole-genome SNP analysis was used to identify genomic patterns and was performed using PhAME (v1.0.2), a pipeline developed at Los Alamos National Labs (LANL). Phylogenetic trees were visualized with iTOL (55). Genomes of *B. cereus*, *B. cereus* bv *anthracis*, *B. thuringiensis*, and *B. anthracis* that were labeled as “complete” in terms of the level of genome assembly and associated metadata were downloaded from NCBI. The downloaded assemblies underwent QUAST analysis to evaluate their quality. Assemblies were selected based on the number of contigs and per-base quality metrics. Only those assemblies with three contigs were chosen for *B. anthracis*, while assemblies with up to four contigs were selected for *B. cereus*, and up to five contigs for *B. thuringiensis*. After analysis, we had 126 *B. cereus*, 19 *B. thuringiensis*, two Bcbva, and 48 *B. anthracis* strains (the accession number and strain names are compiled in Table S1). Gene arrangement was compared between the studied *B. cereus* PATH2418 and known capsule-producing *Bacillus* strains using the Burrows-Wheeler Alignment tool (BWA) (56) and visualized with Mauve.

Capsule operons of *B. cereus* FDAARGOS_802, *B. thuringiensis* BGSC 4C1, and *B. anthracis* Ames Ancestor were downloaded from the NCBI (details entailed in Table S3) and aligned with the capsule operon of *B. cereus* PATH2418 using the NCBI BLASTN tool. The alignment results were visualized using Easyfig 2.2.5, a genome alignment visualization tool (27).

## Data Availability

All data from the study are available in the article. Sequence data have been deposited in publicly accessible databases. The fastq file was submitted to the NIH Sequence Read Archive under BioProject PRJNA1241850, BioSample SAMN47572344, and SRA file SRR32854702. The Flye assembled genome sequences are available in GenBank at accession numbers CP196405-CP196408.

## SUPPLEMENTAL MATERIAL

Supplemental Material spectrum.00899-25-s0001.docx Tables S1 to S4; Fig. S1 and S2.

ASM does not own the copyrights to Supplemental Material that may be linked to, or accessed through, an article. The authors have granted ASM a non-exclusive, world-wide license to publish the Supplemental Material files. Please contact the corresponding author directly for reuse.
