# Supplementary material for: Molecular and structural characterization of a Bacillus cereus strain producing an anthrax-like capsule
Source: Microbiol Spectr. 2025 Aug 13;13(10):e00899-25. doi: 10.1128/spectrum.00899-25 (PMC12502663; doi:10.1128/spectrum.00899-25)
Supplement: Supplemental Material — Tables S1 to S4; Fig. S1 and S2. [file spectrum.00899-25-s0001.docx]

**Table S1**: Accession number and strain name of *Bacillus spp*. that were used for whole-genome SNP-based phylogenetic analysis.

| **Accession Number** | **Name of Strain** | **Accession Number** | **Name of Strain** |
| --- | --- | --- | --- |
| GCA_002277915.1 | *B. anthracis* 14RA5914 | GCA_009739965.1 | *B. cereus* DLOU-Tangshan |
| GCA_006088855.1 | *B. anthracis* 17OD930 | GCA_009739985.1 | *B. cereus* DLOU-Weihai |
| GCA_022221145.1 | *B. anthracis* 188678-1 | GCA_023516415.1 | *B. cereus* DQ01 |
| GCA_000832965.1 | *B. anthracis* 2002013094 | GCA_030518615.1 | *B. cereus* DW444 |
| GCA_000833275.1 | *B. anthracis* 35 | GCA_000239195.1 | *B. cereus* F837/76 |
| GCA_000875715.1 | *B. anthracis* A1144 | GCA_016727405.1 | *B. cereus* FDAARGOS_1084 |
| GCA_022220965.1 | *B. anthracis* A168 | GCA_013267475.1 | *B. cereus* FDAARGOS_780 |
| GCA_022220985.1 | *B. anthracis* A178 | GCA_013267455.1 | *B. cereus* FDAARGOS_781 |
| GCA_022220505.1 | *B. anthracis* A182 | GCA_013267775.1 | *B. cereus* FDAARGOS_797 |
| GCA_022220925.1 | *B. anthracis* A193a | GCA_013267275.1 | *B. cereus* FDAARGOS_798 |
| GCA_022221045.1 | *B. anthracis* A27 | GCA_016027015.1 | *B. cereus* FDAARGOS_918 |
| GCA_000833065.1 | *B. anthracis* Ames | GCA_030067875.1 | *B. cereus* FFI_gr_36 |
| GCA_000008445.1 | *B. anthracis* Ames Ancestor; A2084 | GCA_030067855.1 | *B. cereus* FFI_gr_46 |
| GCA_029636705.1 | *B. anthracis* AN17-14_S4 | GCA_000832525.1 | *B. cereus* FM1 |
| GCA_029636405.1 | *B. anthracis* AN17-384_2 | GCA_000978375.1 | *B. cereus* FORC_005 |
| GCA_029636605.1 | *B. anthracis* AN18-416_2 | GCA_025035865.1 | *B. cereus* FORC_010 |
| GCA_000832665.1 | *B. anthracis* BA1015 | GCA_001518875.1 | *B. cereus* FORC_013 |
| GCA_045038045.1 | *B. anthracis* BA20200413YY | GCA_001721145.1 | *B. cereus* FORC_024 |
| GCA_000295695.2 | *B. anthracis* BF1 | GCA_002220285.1 | *B. cereus* FORC_047 |
| GCA_022221085.1 | *B. anthracis* BF1 | GCA_002214765.1 | *B. cereus* FORC_048 |
| GCA_021390035.1 | *B. anthracis* BF5 | GCA_005707595.1 | *B. cereus* FORC_086 |
| GCA_030166675.1 | *B. anthracis* BHY1401 | GCA_002000005.1 | *B. cereus* FORC021 |
| GCA_022221025.1 | *B. anthracis* BUL 12 | GCA_006384875.1 | *B. cereus* FORC087 |
| GCA_022220625.1 | *B. anthracis* BUL 16 | GCA_003020845.1 | *B. cereus* FORC60 |
| GCA_022220585.1 | *B. anthracis* BUL 19 | GCA_000724585.1 | *B. cereus* FT9 |
| GCA_022220605.1 | *B. anthracis* BUL 31 | GCA_008041975.1 | *B. cereus* G1-1 |
| GCA_043295875.1 | *B. anthracis* Bundesheer | GCA_045780845.1 | *B. cereus* G1-11 |
| GCA_041508535.1 | *B. anthracis* CWH_147 | GCA_000832805.1 | *B. cereus* G9241 |
| GCA_000725325.1 | *B. anthracis* HYU01 | GCA_000021305.1 | *B. cereus* G9842 |
| GCA_042193355.1 | *B. anthracis* IAL 52 | GCA_024498915.1 | *B. cereus* GSICC 30237 |
| GCA_003227955.1 | *B. anthracis* London_499 | GCA_033452345.1 | *B. cereus* GUCC 3 |
| GCA_043191725.1 | *B. anthracis* MH-PR | GCA_013394245.1 | *B. cereus* H2 |
| GCA_043190285.1 | *B. anthracis* MH-VW | GCA_003568565.1 | *B. cereus* HBL-AI |
| GCA_000832505.1 | *B. anthracis* Ohio ACB | GCA_013284505.2 | *B. cereus* HD1_4B |
| GCA_019693235.1 | *B. anthracis* P04210076 | GCA_013284455.2 | *B. cereus* HD2_4 |
| GCA_006742565.1 | *B. anthracis* PCr | GCA_001635995.1 | *B. cereus* HN001 |
| GCA_022014755.1 | *B. anthracis* PNO2 | GCA_019704155.2 | *B. cereus* HT18 |
| GCA_022221225.1 | *B. anthracis* Pollino 3734 | GCA_030064245.1 | *B. cereus* IBA1 |
| GCA_000832745.1 | *B. anthracis* RA3 | GCA_001941905.1 | *B. cereus* ISSFR-3F |
| GCA_000832565.1 | *B. anthracis* SK-102 | GCA_001941885.1 | *B. cereus* ISSFR-9F |
| GCA_001543225.1 | *B. anthracis* Stendal | GCA_021655155.1 | *B. cereus* J10 |
| GCA_001654475.1 | *B. anthracis* Tangail-1 | GCA_021655175.1 | *B. cereus* J39 |
| GCA_022221165.1 | *B. anthracis* Tangail-4/2 | GCA_021655215.1 | *B. cereus* J62 |
| GCA_022220685.1 | *B. anthracis* Tyrol 3520 | GCA_021655235.1 | *B. cereus* J75 |
| GCA_001936375.1 | *B. anthracis* Tyrol 4675 | GCA_001941925.1 | *B. cereus* JEM-2 |
| GCA_022221125.1 | *B. anthracis* UR-1 | GCA_009738575.1 | *B. cereus* JHU |
| GCA_022221345.1 | *B. anthracis* Vollum | GCA_002215175.1 | *B. cereus* K8 |
| GCA_000832445.1 | *B. anthracis* Vollum 1B | GCA_040959295.1 | *B. cereus* L6 |
| GCA_000022505.1 | *B. cereus* 03BB102 | GCA_030406205.1 | *B. cereus* lycx |
| GCA_000832405.1 | *B. cereus* 03BB102 | GCA_002216125.1 | *B. cereus* M13 |
| GCA_000789315.1 | *B. cereus* 03BB87 | GCA_002214725.1 | *B. cereus* M3 |
| GCA_004771155.1 | *B. cereus* 1000305 | GCA_029201265.1 | *B. cereus* M72-4 |
| GCA_016774555.1 | *B. cereus* 21155 | GCA_024299105.1 | *B. cereus* MB1 |
| GCA_028607045.1 | *B. cereus* 2-6A | GCA_004801195.1 | *B. cereus* MH19 |
| GCA_021655255.1 | *B. cereus* 30040 | GCA_002813875.1 | *B. cereus* MLY1 |
| GCA_021655275.1 | *B. cereus* 30043 | GCA_021654875.1 | *B. cereus* MRY14-0045 |
| GCA_021655295.1 | *B. cereus* 30048 | GCA_021654895.1 | *B. cereus* MRY14-0057 |
| GCA_021655335.1 | *B. cereus* 30075 | GCA_021654975.1 | *B. cereus* MRY14-0079 |
| GCA_021655355.1 | *B. cereus* 30077 | GCA_022700995.1 | *B. cereus* N435-1 |
| GCA_000832765.1 | *B. cereus* 3a | GCA_040268315.1 | *B. cereus* NBNZ-2162 |
| GCA_034078685.1 | *B. cereus* A01 | GCA_001277915.1 | *B. cereus* NJ-W |
| GCA_000635895.2 | *B. cereus* A1 | GCA_021442085.1 | *B. cereus* NR1 |
| GCA_006349715.2 | *B. cereus* A22 | GCA_026684175.1 | *B. cereus* NW6 |
| GCA_006349735.2 | *B. cereus* A24 | GCA_027920485.1 | *B. cereus* PL22-16A |
| GCA_016774575.1 | *B. cereus* AFA01 | GCA_025917685.1 | *B. cereus* PT1 |
| GCA_000021225.1 | *B. cereus* AH187 | GCA_000013065.1 | *B. cereus* Q1 |
| GCA_001880305.1 | *B. cereus* AR156 | GCA_041879445.1 | *B. cereus* QKG-2024 |
| GCA_000008005.1 | *B. cereus* ATCC 10987 | GCA_000835185.1 | *B. cereus* S2-8 |
| GCA_000007825.1 | *B. cereus* ATCC 14579 | GCA_009497015.2 | *B. cereus* SB1 |
| GCA_006094295.1 | *B. cereus* ATCC 14579 | GCA_023008425.1 | *B. cereus* SEM-15 |
| GCA_045287585.1 | *B. cereus* ATCC 14579 | GCA_025502485.1 | *B. cereus* SRCM116293 |
| GCA_000832845.1 | *B. cereus* ATCC 4342 | GCA_032595315.1 | *B. cereus* T |
| GCA_032145515.1 | *B. cereus* B126_1 | GCA_045838415.1 | *B. cereus* Ta-LAA-64 |
| GCA_000021205.1 | *B. cereus* B4264 | GCA_003013315.1 | *B. cereus* TG1-6 |
| GCA_031325985.1 | *B. cereus* B6 | GCA_016917795.1 | *B. cereus* VKM B-370 |
| GCA_040210495.1 | *B. cereus* B9 | GCA_013112375.1 | *B. cereus* WPySW2 |
| GCA_027625975.1 | *B. cereus* BC-01 | GCA_030517775.1 | *B. cereus* Z4 |
| GCA_018309125.1 | *B. cereus* BC06 | GCA_004006495.1 | *B. cereus* ZB201708 |
| GCA_018309145.1 | *B. cereus* BC07 | GCA_039954905.1 | *B. thuringiensis* 9_1 |
| GCA_045277855.1 | *B. cereus* BC116 | GCA_021595545.1 | *B. thuringiensis* B13 |
| GCA_018309165.1 | *B. cereus* BC33 | GCA_025947955.1 | *B. thuringiensis* Bt Gxmzu777-1 |
| GCA_025946485.2 | *B. cereus* BC38B | GCA_003054785.2 | *B. thuringiensis* BT62 |
| GCA_964341285.1 | Bcbva CA | GCA_046529345.1 | *B. thuringiensis* BV5 |
| GCA_000143605.1 | Bcbcva CI | GCA_030166715.1 | *B. thuringiensis* DHC4 |
| GCA_024584625.1 | *B. cereus* C-1 | GCA_013267335.1 | *B. thuringiensis* FDAARGOS_793 |
| GCA_002224345.1 | *B. cereus* C1L | GCA_018885365.1 | *B. thuringiensis* GR007 |
| GCA_002290105.1 | *B. cereus* CC-1 | GCA_016070565.1 | *B. thuringiensis* H3 |
| GCA_023094015.1 | *B. cereus* CDHWZ7 | GCA_006151925.1 | *B. thuringiensis* HM-311 |
| GCA_018884185.1 | *B. cereus* CF4-51 | GCA_045057165.1 | *B. thuringiensis* IPPBIOTSUC-1012 |
| GCA_016774535.1 | *B. cereus* CH | GCA_040712275.1 | *B. thuringiensis* JJ1216 |
| GCA_001635955.1 | *B. cereus* CMCC P0011 | GCA_001692675.1 | *B. thuringiensis* KNU-07 |
| GCA_001635915.1 | *B. cereus* CMCC P0021 | GCA_030718705.1 | *B. thuringiensis* L1 |
| GCA_013177495.2 | *B. cereus* CTMA_1571 | GCA_032825435.1 | *B. thuringiensis* PS3 |
| GCA_002214705.1 | *B. cereus* D12_2 | GCA_000161595.1 | *B. thuringiensis* serovar monterrey BGSC 4AJ1 |
| GCA_000832385.1 | *B. cereus* D17 | GCA_001675515.1 | *B. thuringiensis* ST7 |
| GCA_030291955.1 | *B. cereus* D5_B_69 | GCA_000161695.1 | *B. thuringiensis* serovar pulsiensis BGSC 4CC1 |
| GCA_040937925.1 | *B. cereus* DJ29 | GCA_017751245_1 | *B. thuringiensis* Bt-GS57 |
| GCA_009739925.1 | *B. cereus* DLOU-Changhai |  |  |

**Table S2**: Accession number and strain name of *Bacillus spp*. whose PGA encoding plasmids were used for whole-genome SNP-based phylogenetic analysis.

| **Accession Number** | **Name of Strain** | **Accession Number** | **Name of Strain** |
| --- | --- | --- | --- |
| CP029325.1 | *B. anthracis* strain 17OD930 | CP009475.1 | *B. anthracis* strain Pasteur |
| CP010854.1 | *B. anthracis* strain A1144 | AP019733.1 | *B. anthracis* PCr |
| CP001972.2 | *B. anthracis* str. A16 | CP009695.1 | *B. anthracis* strain RA3 |
| CP009979.1 | *B. anthracis* strain Ames (plasmid unnamed2) | AP014835.1 | *B. anthracis* Shikan-NIID |
| CP116897.1 | *B. anthracis* strain AN17-14_S4 | CP009462.1 | *B. anthracis* strain SK-102 |
| CP116891.1 | *B. anthracis* strain AN17-384_2 | CP014178.1 | *B. anthracis* strain Stendal |
| CP116894.1 | *B. anthracis* strain AN18-416_2 | CP015778.1 | *B. anthracis* strain Tangail-1 |
| CP009698.1 | *B. anthracis* strain BA1035 | CP018905.1 | *B. anthracis* strain Tyrol 4675 |
| CP047133.1 | *B. anthracis* str. BF1 | CP009636.1 | *B. cereus* 03BB108 |
| CP126465.1 | *B. anthracis* strain BHY1401 | CP001748.1 | Bcbva CI |
| CP010320.1 | *B. anthracis* strain Canadian_bison | CP053962.1 | *B. cereus* strain FDAARGOS_802 |
| CP145728.1 | *B. anthracis* strain CVCC40202 | CP015177.1 | *B. thuringiensis* serovar alesti strain BGSC 4C1 |
| AP018445.1 | *B. anthracis* CZC5 | CP017574.1 | *B. thuringiensis* strain SCG04-02 |
| CP012521.1 | *B. anthracis* strain Larissa | NC_002146.1 | *B. anthracis* |
| CP029807.1 | *B. anthracis* strain London_499 | CP139446.1 | *B. anthracis* strain OK-05 |

**Table S3**: Name of Strains whose Capsule Operon was Used to Align with *B. cereus* PATH2418 PGA Capsule Operon

| **Species** | **GeneBank Accession No.** | **Operon location** | **Genes in the Operon** |
| --- | --- | --- | --- |
| *B. anthracis* str. 'Ames Ancestor' plasmid pXO2 | AE017335.3 | 52147-56993 | *capB, capC, capA, capD,* and *capE* |
| *B. thuringiensis* serovar alesti strain BGSC 4C1 plasmid pBMB267 | CP015177.1 | 42707-47544 | *pgsC*, *pgsB*, *capA*, *capD* |
| *B. cereus* strain FDAARGOS_802 plasmid unnamed2 | CP053962.1 | 16702-21171 | *pgsB*, *pgsC*, *capA* family protein, *capD* |

**Table S4:** Chemical shifts (ppm) observed for polyglutamate.

| **Polyglutamate** | **α** | **β** | | | **γ** |
| --- | --- | --- | --- | --- | --- |
|  |  | **a** | | **b** |  |
| ^1^H | 4.13 | 2.08 | 1.92 | | 2.35 |
| ^13^C | 57.5 | 30.6 | | | 35.2 |

**Supplementary Figures:**

**Figure S1. Mauve alignment of capsule encoding plasmids in different *Bacillus* spp.** The alignment compares the promoter and operon regions of the PGA encoding genes from *B. cereus* PATH2418 with *B. anthracis* Ames, *Bacillus cereus* bv *anthracis*, and other *B. cereus* strains. Purple color blocks represent the capsule operon and pink color blocks represent the capsule regulator *acpB*.

**Figure S2.** **SNP-based phylogenetic tree constructed from analysis of capsule operon encoding plasmids of different *Bacillus spp*.** The capsule operon containing plasmid of *B. cereus* PATH2418 strain, the pATH1, in this study (in the yellow background), was closely related to the capsule operon encoding plasmids of *B. thuringiensis* BGSC 4C1 (in blue background) and *B. cereus* strains (in green background). pXO2 plasmids of *B. anthracis* are highlighted in red and pBCXO2 of Bcbva is highlighted in purple.
